# Polymerase chain reaction test for diagnosis of infectious uveitis

**DOI:** 10.1186/s40942-023-00465-w

**Published:** 2023-04-12

**Authors:** Sahba Fekri, Ehsan Barzanouni, Shahram Samiee, Masoud Soheilian

**Affiliations:** 1 Department of Ophthalmology, Labbafinejad Medical Center, Shahid Beheshti University of Medical Sciences, Pasdaran Ave., Boostan 9 St, Tehran, Iran; 2 Clinical Research Development Unit of Labbafinejad Medical Center, Shahid Beheshti University of Medical Sciences, Tehran, Iran; 3 Blood Transfusion Research Center, High Institute for Research & Education in Transfusion Medicine, Tehran, Iran

**Keywords:** Infectious Uveitis, Ocular sampling, Polymerase chain reaction (PCR), Uveitis

## Abstract

**Background:**

To study the clinical utility of broad-range real-time Polymerase Chain Reaction (PCR) assay in patients suspected for infectious uveitis and to analyze the clinical relevance.

**Methods:**

Medical records of patients with uveitis were assessed in whom PCR analysis of intraocular fluids was performed between January 2018 and February 2021. Intraocular samples were investigated for cytomegalovirus (CMV), Epstein-Barr virus (EBV), varicella zoster virus (VZV), herpes simplex viruses type 1 and 2 (HSV_1,2_), human T-lymphotropic virus type 1 (HTLV-1), Toxoplasma gondii and also for bacterial 16 S and fungal 18 S/28S ribosomal DNA (rDNA).

**Results:**

Aqueous paracentesis and vitreous sampling was done for 151 (81.2%) and 35 (18.8%) patients, respectively. Most of the patients had panuveitis (61.3%). PCR results were positive in 69 out of 186 patients (37%) according to the following order: CMV (18 cases), VZV (18 cases), fungal 18s/28s rDNA (17 cases), HSV (9 cases), bacterial 16s rDNA (3 cases), HTLV-1 (2 cases), and Toxoplasma gondii (2 cases). PCR positivity rate was 5.8% in patients with undifferentiated panuveitis. EBV was not detected at all. Initial treatment was changed in 38 patients (20%) based on PCR results. The overall sensitivity, specificity, positive predictive value (PPV), and negative predictive value (NPV) of PCR test for aqueous samples was 82%, 91%, 96%, and 87%, respectively. No significant adverse effect related to sampling was reported.

**Conclusion:**

PCR analysis of intraocular fluids in patients with suspected infectious uveitis plays an important role in confirming diagnosis or changing treatment with good predictive value. However, routine PCR test in patients with undifferentiated panuveitis in order to rule out possible underlying infectious etiology had low benefit.

**Supplementary Information:**

The online version contains supplementary material available at 10.1186/s40942-023-00465-w.

## Key message of the Article

**What is already known on this topic:**

PCR analysis of intraocular fluids in patients with suspected infectious uveitis is a useful adjunct method.

**What this study adds:**

PCR test has low clinical utility in patients with undifferentiated panuveitis and low suspicion for infectious etiology. Multicenter studies is needed for better judgment.

**How this study might affect research, practice or policy:**

These findings suggest that developing an algorithm with clear indications for considering PCR analysis in patients with uveitis may improve its diagnostic value.

**Supplementary Information:**

The online version contains supplementary material available at 10.1186/s40942-023-00465-w.

## Introduction

One of the most important steps in approach to a patient with uveitis is to distinguish infectious from non-infectious etiologies as they may present with similar clinical features but essentially needs different management. The prevalence of infectious uveitis significantly varies between developed and developing countries. It accounts for less than 20% of uveitis in the United States but its prevalence reaches to 30–50% in developing countries [1–3]. In Iran, infectious uveitis accounts for 16.5–23.5% of all cases of uveitis and the most common cause is toxoplasmosis [4]. Although infectious uveitis usually presents as posterior and panuveitis, but the role of viral infections especially human herpes viruses (HHVs) as the etiology of anterior uveitis has become more prominent recently with the advent of polymerase chain reaction (PCR) analysis of aqueous humor [5].

PCR is a laboratory technique which amplifies a very small sample of deoxyribonucleic acid (DNA) or Ribonucleic acid (RNA) to numerous copies. This ability can be used for detection of pathogens from intraocular fluids. Its high accuracy and reproducibility, safety and fastness, and working with small quantity of samples has made PCR a useful method for diagnosing infectious uveitis [6, 7].

The purpose of this study was to assess the clinical utility of PCR test in patients with suspected infectious uveitis, and its impact on patients’ management.

## Methods

This is a retrospective, chart review study conducted at a referral university hospital, Tehran, Iran. Medical records of patients with uveitis who underwent intraocular fluid sampling for PCR test, between January 2018 and February 2021, were analyzed. This study was adhered to the tenets of Declaration of Helsinki and was approved by ethical committee of Shahid Beheshti University of Medical Sciences (Reference number: IR.SBMU.MSP.REC.1400.478). Informed written consent was obtained from all patients.

Demographics of the patients, medical history, slit-lamp and fundus examination findings, intraocular pressure (IOP), anatomical location of inflammation (in accordance with the SUN classification) [8], initial clinical diagnosis (prior to sampling), disease course, treatment modality, type of ocular fluid specimen, PCR results, and post procedure management were extracted from the charts. Cases with incomplete data or lost to follow-up were excluded from the study.

Intraocular fluid sampling was performed at operating room under sterile condition. After instillation of topical anesthesia, standard preparation with povidone iodine 5%, and lid speculum placement, a 30 gauge half inch needle was used for anterior chamber paracentesis. At least 0.1 ml of aqueous was extracted for PCR analysis. Vitreous sampling was done during diagnostic pars plana vitrectomy. Specimens were transported on ice pack to an authorized laboratory for PCR analysis on the same day of sampling.

### PCR technique

All samples were kept in -70 °C before extraction procedure. DNA extraction was done by Qi amp DNA blood mini kit (Qiagen, Germany) according manufacturer procedure. A plasmid containing fragment of Bromo Mosaic Virus (BMV) was used as internal control and extraction checking. Briefly 200 µl of sample mixed with lysing buffer and IC. After incubation and several washing steps, purified DNA preserved in -70 °C before initiation of PCR procedure.

A broad-range real-time PCR technique was used for detection of cytomegalovirus (CMV), Epstein-Barr virus (EBV), varicella zoster virus (VZV), herpes simplex viruses type 1 and 2 (HSV1,2), human T-lymphotropic virus type 1 (HTLV-1), Toxoplasma gondii and also for bacterial 16 S and fungal 18 S/28S ribosomal DNA (rDNA). We designated all primers and probes and passed both Insilco and experimental validation. Oligo’s were synthesized by Metabion company (Germany). Real-time assay was done by quantitect probe PCR kit (Qiagen, Germany) as basic master mix with a combination of each primer probe set of interested organism and internal control according to manufacturer recommendations. The real-time PCR was performed on Rotor-Gen Q platform. Positive and negative controls for each pathogen were included. Quantitative data analysis was performed by Rotor-Gen Eqs. 2-3-4-3 version software.

### Statistical analysis

To present data, we used mean, standard deviation (SD), range, frequency and percentage. We used the Chi-square or Fisher exact test for statistical analysis; P value less than 0.05 was considered as statistically significant. All statistical analyses were performed by SPSS (IBM Corp. Released 2013. IBM SPSS Statistics for Windows, Version 22.0. Armonk, NY: IBM Corp., USA).

To calculate sensitivity and specificity of PCR test, final clinical diagnosis during follow-up was determined as gold standard. If positive or negative PCR results were consistent with final diagnosis, they considered to be true. When clinical presentations, course of disease, response to medications and final outcome were against the recovered pathogen by PCR test, then it labeled as false positive. False negative results included the cases that proved to have infectious uveitis during follow-up but PCR test did not detect the related DNA.

## Results

Data of 186 patients were analyzed. The mean age of the patients was 48.81 ± 17.86 (range, 5–86) years. There was no sex predominance. Majority of patients were immunocompetent (73.6%) and 4.8% of patients had history of substance abuse. Unilateral ocular inflammation was far more common than bilateral involvement (83% vs. 17%). Most of the patients (75%) underwent intraocular sampling during acute phase of inflammation (less than 3 months’ duration). Only 44 patients (24%) did not use any medications at the time of sampling. Others (76%) were under antibiotic, antiviral or anti-inflammatory therapy. Persistent inflammation despite proper immunomodulatory therapy (IMT), uncontrolled IOP with medication, suspected viral etiology in anterior uveitis, atypical presentations especially in immunocompromised patients, clinical features overlap between different pathogens and undifferentiated panuveitis were the main reasons for sampling. Aqueous paracentesis and vitreous sampling was done for 151 (81.2%) and 35 (18.8%) patients, respectively. No ocular complication was reported related to intraocular fluid sampling.

Baseline ocular features of the patients are shown in Table 1. Panuveitis was the most common (61%) and intermediate uveitis was the least common (1%) type of intraocular inflammation at presentation. More than 70% and 90% of the patients had vitreous and anterior chamber (AC) inflammation respectively.

**Table 1:** Ocular presentations of the patients at the time of sampling (N = 186)

*Laterality of uveitis*,	
*Unilateral*,	
*Right eye*	*68(36%)*
*Left eye*	*86(46%)*
*Bilateral*	*32(18%)*
*Anatomical distribution of uveitis*,	
*Anterior uveitis*	*34(18%)*
*Intermediate uveitis*	*2(1%)*
*Posterior uveitis*	*36(19%)*
*Panuveitis*	*114(62%)*
*Lens status*,	
*Phakic*	*123(66%)*
*Pseudophakic*	*63(34%)*
*Ophthalmic examination findings*,	
AC inflammation	171(92%)
Keratic precipitates	78(42%)
Hypopion	31(17%)
Iris atrophy	10(5%)
Posterior synechia	15(8%)
Elevated IOP	42(23%)
Vitritis	135(72%)
Optic disc involvement*	15(8%)
Retinitis*	83(45%)
Vasculitis*	60(33%)
Choroiditis*	18(10%)

AC, anterior chamber; IOP, intraocular pressure

* These findings were evaluated only in patients with visible posterior segment.

Panuveitis, CMV retinitis and endogenous endophthalmitis were among the most common pre-sampling clinical diagnoses. The inability to perform reliable fundus examination due to dense vitritis, and importance of timely diagnosis of infectious etiology in immunosuppressed patients had significant role in decision making for sampling. Presumed ocular tuberculosis was not reported in any patient.

PCR results were positive in 69 out of 186 cases (37%). The frequency ranged from zero for intermediate uveitis up to 47% for panuveitis (Table 2). CMV and VZV were the most common recovered pathogens (9.7%). HSV1 and HSV2 was detected in 9 cases (4.8%), Toxoplasma gondii in 2 cases, and HTLV-1 in two cases. No case was positive for EBV. HSV and 18s rDNA (specific for fungal infection) were the only pathogens found in cases with anterior uveitis; the latter was due to specimen contamination or technical error. However, concomitant positive results for VZV and CMV was seen in two cases with panuveitis.

**Table 2:** Polymerase chain reaction (PCR) results for each pathogen based on location of inflammation

*Pathogen*	*Anatomical Classification of Uveitis*				
	*Anterior Uveitis (N = 34)*	*Intermediate Uveitis (N = 2)*	*Posterior Uveitis (N = 36)*	*Panuveitis* *(N = 114)*	*Total* *(N = 186)*
*VZV*	*0*	*0*	*1*	*17*	*18*
*CMV*	*0*	*0*	*1*	*17*	*18*
*HSV*	*4*	*0*	*0*	*5*	*9*
*HTLV-1*	*0*	*0*	*1*	*1*	*2*
*Toxoplasma gondii*	*0*	*0*	*2*	*0*	*2*
*Bacteria*	*0*	*0*	*0*	*3*	*3*
*Fungus*	*4*	*0*	*2*	*11*	*17*
*Positive results (%)*	*8/34 (23.5%)*	*0/2 (0%)*	*7/36 (19.5%)*	*54/114 (47%)*	*69/186 (37%)*

*VZV, varicella zoster virus; CMV, cytomegalovirus; HSV, herpes simplex virus; HTLV, human T-lymphotropic virus 1*

Distribution of PCR results based on initial clinical diagnosis is demonstrated in Table 3. Of 19 patients with anterior uveitis who were clinically suspected for viral etiology, 3 cases were positive for HSV. No viral pathogen was found in 10 patients with Fuchs uveitis syndrome (FUS) and Posner-schlossman syndrome (PSS). About 68% of cases with clinical diagnosis of necrotizing herpetic retinitis and 50% of cases with diagnosis of CMV retinitis were positive for VZV and CMV, respectively. Although HSV1,2 were not detected in cases with necrotizing herpetic retinitis, but they were found in 2 cases of panuveitis and 4 cases of suspected bacterial endogenous endophthalmitis which led to the change of initial treatment. PCR analysis of aqueous humor became positive for Toxoplasma gondii in two patients with pre-sampling clinical diagnosis of atypical ocular toxoplasmosis. One case of idiopathic retinal vasculitis with multiple recurrences during steroid tapering and one case of panuveitis were found to be positive for HTLV-1. More than 94% of PCR results in patients with undifferentiated panuveitis were negative, mainly contributed to the large number of overall negative results. About 76% of positive results (13 out of 17) for 18s rDNA (fungal infection) were compatible with initial and final diagnosis, others were false positive, confirmed during follow up.

**Table 3:** Distribution of polymerase chain reaction (PCR) results based on pretest clinical diagnosis

Initial diagnosis	Site of sampling		PCR results								
	Aqueous	Vitreous	Negative	HSV	VZV	CMV	EBV	HTLV-1	Toxoplasma	Bacteria 16 S	Fungal 18 S
Viral anterior uveitis	19	0	14	3	0	0	0	0	0	0	2
Viral etiology in Fuchs uveitis syndrome	8	0	6	0	0	0	0	0	0	0	2
Viral etiology in Posner-Schlossman syndrome	2	0	2	0	0	0	0	0	0	0	0
Necrotizing herpetic retinitis	19	3	3	0	15	4	0	0	0	0	0
CMV retinitis	22	6	9	0	3	14	0	0	0	0	2
Toxoplasma retinochoroiditis	2	0	0	0	0	0	0	0	2	0	0
Undifferentiated panuveitis	45	7	49	2	0	0	0	1	0	0	0
Retinal vasculitis	1	0	0	0	0	0	0	1	0	0	0
Chronic post-operative endophthalmitis	10	2	10	0	0	0	0	0	0	0	2
Bacterial endogenous endophthalmitis	15	13	22	4	0	0	0	0	0	1	1
Fungal endogenous endophthalmitis	8	4	2	0	0	0	0	0	0	2	8

The impact of PCR test on the management of patients is shown in Table 4. PCR results made change in initial treatment of 38 patients (20%). Table 5 shows the overall sensitivity, specificity, positive predictive value (PPV), and negative predictive value (NPV) of PCR test for aqueous samples.

**Table 4:** Distribution of treatment change after Intraocular PCR results

Change in initial treatment (%)	38 (20%)
Continuation of initial treatment (%)	101 (55%)
Dose modification of prescribed drugs or initiation of adjuvant therapy (%)	47 (25%)

**Table 5:** Sensitivity, Specificity, PPV, NPV of PCR test for aqueous samples

No. of Patients	True Positive results	False Positive results	True Negative results	False Negative Results	Sensitivity	Specificity	PPV*	NPV^¥^
151	55	2	82	12	82%	91%	96%	87%

* PPV: Positive Predictive Value

¥ NPV: Negative Predictive Value

## Discussion

This is the first study from Iran (the Middle East), reporting the results of PCR analysis of intraocular fluids in patients suspected for infectious uveitis. Using broad-range real-time PCR technique, the positivity rate was 37% and the most common detected pathogens were VZV and CMV. The overall sensitivity and specificity of PCR test for aqueous samples was 82% and 91%, respectively. The results are comparable with studies used multiplex PCR (85.2% and 97.8%) [9], comprehensive PCR (91.3% and 98.8%) [10], and uniplex PCR (90.2% and 93.9%) [11]. It is noteworthy to mention that these values are not representative of real diagnostic value of PCR test and seems to be underestimated as this technique usually has been performed in patients clinically suspected of infectious uveitis.

More than three decades has been passed since introduction of PCR method as a useful adjunct for the diagnosis of infectious uveitis. Many studies have evaluated its safety and utility with different results. This diversity among studies is related to many factors including study design, study population, anatomical location of uveitis, ethnics, epidemiological and geographical factors affecting the distribution of infectious etiologies, site of sampling, indications, immune status of the patients, type of PCR technique, and even laboratory experience.

Although PCR test is performed for all types of uveitis, its main utility is in the posterior and panuveitis. This issue can be explained by higher prevalence of infectious etiologies in conjunction with more difficulty in reliable fundus examination in these entities. Overall, 114 out of 186 cases (61.3%) had panuveitis, nearly half of them (47%) had also positive PCR results. Iran is located along ancient “Silk Road” and Behcet disease (BD) and Vogt-Koyanagi-Harada (VKH) syndrome are among the most prevalent known causes of panuveitis [4]. In this study, panuveitis with negative PCR result was seen in three main groups of patients. First, the patients with presenting signs of panuveitis who were finally diagnosed to have BD or VKH syndrome following thorough work-up and disease course. Second, known cases of Behcet disease under immunomodulatory therapy (IMT) who presented with new retinitis lesions and vasculitis during follow-up. In this group of patients, PCR test was done to rule out superimposed infectious necrotizing retinitis including CMV retinitis before IMT escalation. Third, the patients categorized to have undifferentiated panuveitis including patients with unknown etiology or the cases with ocular signs of BD uveitis who did not fulfill the International Study Group (ISG) criteria [12]. Viral pathogens (HSV and HTLV-1) were found only in 3 patients with undifferentiated panuveitis (less than 6%) after PCR test. These findings suggest low diagnostic value of PCR assay in patients with undifferentiated panuveitis when infectious etiology is less probable. In contrast, Fallon et al. reported the significant role of PCR analysis in uveitis patients of unknown etiology [13]. However, they also showed when pre-sampling probability of positive PCR result was low, based on clinician impression, PCR would not help. Multicenter studies is needed for better judgment.

PCR analysis plays even more prominent role in immunocompromised patients because these cases have frequently atypical presentations and unpredictable production of antibodies. PCR is preferred in diagnosis of viral infections in comparison with Goldmann-Witmer coefficient (GWC) analysis of aqueous humor in immunocompromised patients [14]. In populations with high prevalence of human immunodeficiency virus (HIV) infection, early and routine intra-ocular fluid PCR test in suspected patients is recommended [15]. Harper et al. showed that patients with possible infectious posterior uveitis, especially those with vascular or optic nerve inflammation, extensive retinitis, or immunocompromised state may benefit from adjunctive anterior chamber PCR testing [16]. Moreover, the majority of patients in whom PCR results led to initial treatment change were immunocompromised (85%). In our study, less than one third of patients were immunocompromised and PCR showed satisfactory results for confirmation of CMV retinitis while clinical presentations were inconclusive.

As mentioned before, geographical distribution may influence PCR outcomes. A retrospective cohort study from Indonesia evaluated 87 patients suspected of infectious uveitis [17]. Most of them had panuveitis (45%) and vitreous samples were taken from patients undergone diagnostic vitrectomy (8%). The PCR positivity rate was 17.2% and Mycobacterium tuberculosis (MTB) and Toxoplasma gondii were the most frequent observed microorganisms. Moreover, the detection rate of MTB was higher from vitreous samples in contrast to Toxoplasma gondii which was detected only from aqueous samples. The authors concluded that their findings were consistent with the fact that 68% and 20% of all infectious uveitis in Indonesia was caused by toxoplasmosis and tuberculosis, respectively. In another retrospective, interventional study from South India, posterior uveitis was the most prevalent type (38%) followed by anterior uveitis (34%) [11]. MTB was the most ordered test, with a PCR sensitivity of 71.4% and specificity of 76.8%. Although the overall sensitivity and specificity of PCR analysis was 90.2% and 93.9%, respectively, PCR results were negative for all 5 cases of toxoplasmosis. These findings differ largely with those reported on other studies from United States [13] and South Korea [18] in which VZV and CMV were the most frequent identified pathogens by PCR, similar to our study. Based on previous report from our center, TB-associated uveitis has been found in 1.5% of patients [4]. In the present study, MTB was not considered in PCR analysis because there was no patient suspected of TB-associated uveitis. Two patients had atypical presentations of ocular toxoplasmosis that PCR analysis of aqueous humor confirmed the diagnosis in both of them. It is still controversial which site of sampling is superior in ocular toxoplasmosis. Wide range of positive results from aqueous samples have been reported (0% up to 100%) [19–22]; Nevertheless, Bodaghi and his colleagues showed that vitreous samples yield better results [23].

In a study from Switzerland, Chronopoulos et al. evaluated the safety and utility of aqueous humor PCR in uveitis [24]. Records of 45 patients were analyzed for common viral pathogens and Toxoplasma gondii. The overall PCR positivity was about 50% and therapy was changed in 38% of patients based on PCR results. The distribution of patients with anterior and posterior uveitis was fairly equal (40%) and 86% of patients with hypertensive anterior uveitis were positive for HSV, VZV, and CMV including remarkable number of patients with clinical diagnosis of FUS and PSS. However, the outcome of PCR testing in anterior uveitis is not always promising. Anwar and colleagues performed PCR analysis for 53 patients with suspected infectious anterior uveitis [25]. Overall, 13% of patients had a change in management, showing relatively low diagnostic utility of PCR in this type of uveitis. In current study, 8 out of 34 patients (23.5%) with anterior uveitis had positive results and HSV was the mere viral pathogen detected. No viral etiology was found in patients with FUS or PSS. Excluding patients who were falsely positive for panfungal 18s rDNA, treatment was changed in 12% of patients. All false-positive results belonged to a limited time period, supposed to be related to contamination or technical fault.

Two cases of HTLV-1 was detected in our study. Although most patients with HTLV-1-associated uveitis usually present with intermediate uveitis [26, 27], panuveitis and posterior vasculitis were the prominent features in our patients. In fact, HTLV-1-related uveitis is an example of uveitides which previously classified as idiopathic uveitis, but with the advent of PCR analysis, it has been identified as a new clinical entity. Interestingly, there is an assumption that the infected intraocular CD4 + T cells with HTLV-1 are responsible for the release of viral genome during inflammation [28].

Vitreous samples were obtained by pars plana vitrectomy and endogenous endophthalmitis was the main pre-sampling impression. Three hospitalized patients with endogenous endophthalmitis secondary to complicated pyelonephritis and prostatitis, improved dramatically after combination therapy with antibiotics and antifungal drugs, based on concurrent positive PCR results for panbacterial and panfungal genome.

PCR analysis of intraocular fluids may also lead to change in treatment plan. In a study included all classes of uveitis similar to our study, therapeutic regimen was changed in 37% of patients after PCR analysis; this value was 20% in patients with posterior uveitis and 24% in cases with anterior uveitis [24].

Anterior chamber paracentesis seems to be a very safe procedure in uveitis patients with no long-term complications [29, 29]. We also did not find any documented complication in patient records.

Our study had some limitations. It was a retrospective study performed in a referral university hospital located in the capital of Iran with possible selection bias. Multicenter studies with larger sample size are needed for better description of population. There was no specific criteria for considering PCR test. A significant number of patients (76%) were using various types of medications prior to sampling. So, the effect of these medications on PCR results is unclear.

## Conclusion

PCR test is a safe, rapid, and sensitive assay for diagnosis of infectious uveitis in conjunction with clinical exam, especially in cases with atypical presentations or unsatisfactory therapeutic responses. Beside its advantages, routine order of PCR test in patients with undifferentiated panuveitis and low probability for infectious etiology seems worthless. Multicenter studies is needed for better judgment. Moreover, its usage for confirmatory purposes when clinical presentations is compatible with infectious uveitis should be assessed in cost-effectiveness studies. Developing an algorithm with clear indications for considering PCR analysis in patients with uveitis may improve its diagnostic value.

## Electronic supplementary material

Below is the link to the electronic supplementary material.

Supplementary Material 1

## Data Availability

The datasets are available from the corresponding author upon reasonable request.
