# Supplementary material for: Polymerase chain reaction test for diagnosis of infectious uveitis
Source: Int J Retina Vitreous. 2023 Apr 12;9:26. doi: 10.1186/s40942-023-00465-w (PMC10091687; doi:10.1186/s40942-023-00465-w)
Supplement: Supplementary file 1 — Supplementary Material 1 [file 40942_2023_465_MOESM1_ESM.docx]

***Dear Editor-in-chief***

*The authors would like to thank the reviewers for their time and their valuable comments on Evaluation of our proposed Original Article entitled, "* *Polymerase Chain Reaction test for diagnosis of infectious uveitis".* We hope to find the arguments convincing.

All changes are highlighted in yellow.

**Reviewer # 3 comments with answers:**

**Comment 1.** When the authors present Sensitivity, Specificity, PPV and NPV, they are considering the clinical exam as the gold standard and aim to evaluate the use of PCR as diagnostic method. It is not clear what were the criteria to determine true positives and negative results? The purpose states that patients with suspected infectious uveitis were included but how did the authors differentiate true positive and true negative infectious uveitis?

*Answer:* The method for calculating sensitivity and specificity of PCR test is almost the same in previously published articles that evaluated the utility of PCR assay in infectious uveitis. Similarly, we considered final clinical diagnosis during follow-up as gold standard, which was based on clinical presentations, disease course, response to medications and final outcome.

However, clear explanation was added to the manuscript (last paragraph of methods, prior to result section).

**Comment 2.** The false positive values could be associated with a non-infectious uveitis so that a negative result on PCR is not related to a poor performance of the technique but just an inappropriate indication for the test.

*Answer*: This statement is completely true. In the present study, the overall sensitivity, specificity, positive predictive value (PPV), and negative predictive value (NPV) of PCR test for aqueous samples was 82%, 91%, 96%, and 87%, respectively. These findings confirm that PCR test is a valuable diagnostic tool in patients with suspected infectious uveitis. As you mentioned, we concluded that routine PCR test in patients with undifferentiated panuveitis in order to rule out possible underlying infectious etiology had low benefit and we suggested that developing an algorithm with clear indications for considering PCR analysis in patients with uveitis may improve its diagnostic value.

We changed the diagnostic utility of PCR test to clinical utility for better conception.

**Comment 3.** Table 4 is not necessary as these numbers are already in the text.

*Answer:* You are right. As presenting results in tables is more suitable and informative than text, we decided to remain the table and revise the text.

**Comment 4**. I would recommend to revise the study purpose so that instead of evaluating the PCR diagnostic utility you would simply evaluate the PCR results in patients with suspected infectious uveitis. You cannot draw conclusion regarding a diagnostic tool if the true positive and negatives values are not clearly defined nor the proper indication for a test is followed.

*Answer:* The revision was made.
